# Supplementary material for: Results of the 2020 fastMRI Challenge for Machine Learning MR Image Reconstruction
Source: IEEE Trans Med Imaging. Author manuscript; Available in PMC 2021 Sep 9. (PMC8428775; doi:10.1109/TMI.2021.3075856)
Supplement: supp1-3075856 [file NIHMS1737265-supplement-supp1-3075856.pdf]

Supplementary Material for Results of the 2020 fastMRI Challenge for Machine Learning MR Image Reconstruction

Matthew J. Muckley*, Bruno Riemenschneider*, Alireza Radmanesh, Sunwoo Kim, Geunu Jeong, Jingyu Ko, Yohan Jun, Hyungseob Shin, Dosik Hwang, Mahmoud Mostapha, Simon Arberet, Dominik Nickel, Zaccharie Ramzi, Philippe Ciuciu, Jean-Luc Starck, Jonas Teuwen, Dimitrios Karkalousos, Chaoping Zhang, Anuroop Sriram, Zhengnan Huang, Nafissa Yakubova, Yvonne W. Lui, and Florian Knoll

April 23, 2021

Here we include supplementary material for the paper, "Results of the 2020 fastMRI Challenge for Machine Learning MR Image Reconstruction."

1 Summary of Data Statistics

Table 1 shows a summary of the data used in the challenge. The Siemens data set was previously reported in an update of the fastMRI arXiv paper [1]. The GE data set and Philips data sets were collected specifically for the challenge.

All T2 and FLAIR Siemens scans used turbo spin echo. The T1 and T1POST Siemens scans used either turbo spin echo or FLASH. The T2 and FLAIR Philips scans used turbo spin echo. The T1 Philips scans used either spin echo or fast field echo. All GE scans used fast spin echo, i.e., were derivatives of their sequence type "FSE-XL". The T1 and T1POST GE scans were of type FLAIR.

2 Analysis of Background Masking

The HungryGrads submitted to all tracks and received a very low SSIM score between 0.4 and 0.5. The reason for the low score was setting the background to all 0s. We performed a post-hoc analysis where we masked the challenge set prior to calculating SSIM. The challenge set was small enough as to admit manual inspection of the masks. The results are shown in Figure S1. The use of masking substantially improved the scores of HungryGrads. However, even with the use of masking, they would not have been a finalist in any of the tracks.

References

[1] J. Zbontar, F. Knoll, A. Sriram, T. Murrell, Z. Huang, M. J. Muckley, A. Defazio, R. Stern, P. Johnson, M. Bruno, M. Parente, K. J. Geras, J. Katsnelson, H. Chandarana, Z. Zhang, M. Drozdzal, A. Romero, M. Rabbat, P. Vincent, N. Yakubova, J. Pinkerton, D. Wang, E. Owens,

Table 1: Summary of Challenge Data

Parameter	T1	T1POST	T2	FLAIR
**Siemens Data Set**				
Field of View (mm)	220×178 - 240×240	220×178 - 240×240	220×165 - 230×230	200×162 - 230×230
Matrix Size	320×260 - 320×330	320×260 - 320×320	320×240 - 384×384	256×208 - 512×512
Slice Thickness (mm)	5	3-5	5	3-5
Number of Slices	2-16	10-16	10-16	12-16
TR (ms)	250-786	247-786	4000-15810	9000
TE (ms)	2-9	2-11	102-115	76-126
Number of Coils	2-24	2-24	2-28	2-44
**GE Data Set**				
Field of View (mm)	220×220 - 240×240	220×220 - 240×240	220×220 - 240×240	220×220 - 240×240
Matrix Size	300×300	300×300	256×256 - 320×320	256×256 - 320×320
Slice Thickness (mm)	5	5	5	5
Number of Slices	8-17	8-17	10-19	8-19
TR (ms)	2885-3268	2884-3268	3112-8400	3137-8400
TE (ms)	24-28	24-28	95-112	87-107
Number of Coils	12-19	12-19	12	12
**Philips Data Set**				
Field of View (mm)	229×183 - 224×224		229×182 - 223×223	229×186 - 224×224
Matrix Size	256×204 - 320×320		248×248 - 384×384	248×248 - 320×320
Slice Thickness (mm)	4-5		4-5	4-5
Number of Slices	15-19		15-23	15-27
TR (ms)	276-599		3526-6000	9000-11000
TE (ms)	3-10		90-110	90-120
Number of Coils	12-14		12-32	12-32

C. L. Zitnick, M. P. Recht, D. K. Sodickson, and Y. W. Lui, "fastMRI: An open dataset and benchmarks for accelerated MRI," *arXiv preprint arXiv:1811.08839*, 2018.

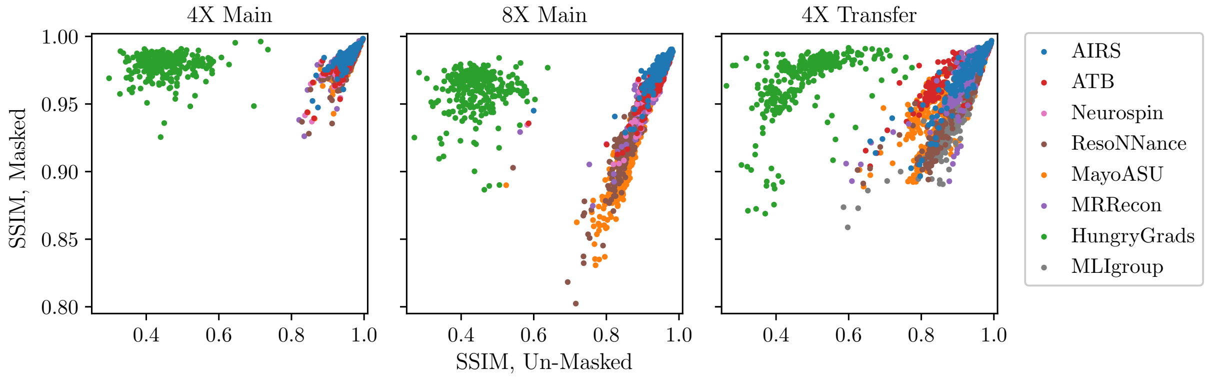

Figure S1: Overview of the impact of a masking procedure. Shown are SSIM scores incorporating masking vs. SSIM scores with no masking. Both methods used the RSS ground truth. 6 outlier points with very low SSIM on both axes were cut off for presentation in the "4X Transfer" plot.
